# Delayed Solvent–Nonsolvent Demixing Preparation and Performance of a Highly Permeable Polyethersulfone Ultrafiltration Membrane

**DOI:** 10.3390/membranes13010039

**Published:** 2022-12-28

**Authors:** Pfano Tshindane, Bhekie B. Mamba, Machawe M. Motsa, Thabo T. I. Nkambule

**Affiliations:** Institute for Nanotechnology and Water Sustainability (iNanoWS), College of Science, Engineering and Technology (CSET), University of South Africa, Florida Science Campus, Private Bag X6, Florida 1709, South Africa

**Keywords:** phase inversion, delayed solvent–nonsolvent demixing, hydrophilicity, fouling

## Abstract

Membrane performance optimization is a critical preparation step that ensures optimum separation and fouling resistance. Several studies have employed additives such as carbon and inorganic nanomaterials to optimize membrane performance. These particles provide excellent results but are rather costly, unstable and toxic to several biological organs. This study demonstrated that performance enhancement can also be achieved through delayed solvent–nonsolvent demixing during phase inversion membrane preparation. The rate of solvent–nonsolvent demixing was delayed by increasing the concentration of the solvent in the coagulation bath. This study employed synthetic and real water samples and several analytical techniques to compare optimized performances and properties of membranes prepared in this study with that of nanoparticle-embedded membranes in the literature. Pure water flux and BSA rejection of the membranes prepared in this study were comparable to those of nanoparticle embedded membranes. This study also shows the influence of delayed solvent–nonsolvent demixing on membrane properties such as morphology, wettability, surface roughness and porosity, thereby showing the suitability of the technique in membrane optimization. Furthermore, fouling studies showed that membranes prepared in this study have high flux recovery when fouled by humic acid feed water (>95%) and above 50% flux recovery with real water samples.

## 1. Introduction

Ultrafiltration (UF) membranes are a class of pressure-driven membranes that have proven to be effective in producing quality drinking water. Ultrafiltration membranes are multifunctional and able to remove persistent natural organic matter (NOM), emerging micropollutants (EMPs) and persistent parasites such as cryptosporidium in a single step [1]. Significant removal of EMPs through UF is achieved when the membrane is optimized to maximize adsorption and retainment of low-molecular-weight molecules. Otherwise, pressure-driven membrane processes that generally achieve significant removal of EMPs are nanofiltration and reverse osmosis [2]. These processes have a high energy demand and are therefore costly for undeveloped countries and communities. Sheng et al. [3] studied the effect of UF post coagulation–flocculation and reported enhanced removal of EMPs. For example, UF enhanced the removal of carbamazepine by 74%, cotinine (100%), metoprolol (82%), triclosan (96%), diclofenac (73%) and sulfamethoxazole (60%). This enhanced removal of EMPs through UF membranes was attributed to a specifically designed UF membrane, complexation of EMPs with NOM during coagulation and subsequent size exclusion and adsorption by the UF membrane.

The performance of UF membranes regarding permeability, removal of low-molecular-weight substances and fouling resistance can be optimized during phase inversion preparation by the addition of additives in the polymer solution. Several studies have used inorganic and carbon nanoparticles as additives in the polymer solution to enhance the removal of dissolved organic matter (DOM) and fouling resistance. Several studies used ZnO and multi-walled carbon nanotubes (MWCNT) to enhance membrane permeability and flux recovery [4,5]. The increased performance (flux: >400 L·m^−2^·h^−1^; flux recovery: >90%) was mainly attributed to increased surface hydrophilicity and effective area. Other studies recorded greater than 90% removal of bovine serum albumin (BSA) by membranes incorporated with graphene oxide (GO) [6,7]. Besides the excellent performance enhancement on membranes, nanoparticles are costly, toxic and have been proven to leach out during filtration [8]. It is these drawbacks that still hinder the large-scale application and commercialization of nanoparticle embedded membranes.

Phase inversion is a common method for UF membrane preparation whereby a polymer is converted from a liquid to a solid state through solvent–nonsolvent demixing and precipitation. Some advantages of phase inversion include ease of functionalization and ability and ease of membrane morphological change. The latter is crucial for significant membrane performance since different membrane morphologies only suit certain operations [9]. Strathmann et al. [10] discussed the thermodynamic aspects and outcomes of a rapid and delayed solvent–nonsolvent demixing process through a ternary phase diagram. Their study showed that delayed demixing produces a sponge-like membrane morphology that generally results in high salt rejections and low water permeability while rapid demixing produces membranes with large finger-like macrovoids in the cross-section. Such membranes generally produce low salt rejections and high water permeabilities [9]. The rate of solvent–nonsolvent demixing also has an effect on membrane surface hydrophilicity and roughness [11,12]. It is therefore possible, cost-effective and environmentally friendlier to optimize membrane performance through rates of solvent–nonsolvent demixing. This work demonstrates that equally high-performance simple polymeric membranes can be prepared through the tuning of the phase separation process and also reinforces that membrane structure and properties dominantly influence mass transport.

Delayed rates of phase inversion through the addition of a solvent in the coagulation bath were used to prepare high-performing and novel pure polymeric membranes. This work also shows that delayed solvent–nonsolvent demixing can produce membranes with high performances comparable to that of nanoparticle embedded membranes which have proven to leach out toxic nanoparticles during operation [13]. This work further demonstrates the effectiveness of backwashing on flux recovery after one cycle of operation. In addition, surface and cross-section properties of the membranes were studied using scanning electron microscopy (SEM), atomic force microscopy (AFM) and contact angle measurements.

## 2. Materials and Methods

### 2.1. Materials

Polymer solutions were prepared using polyethersulfone (PES) as a polymeric material, polyethylene glycol (PEG) BioUltra 400 Da and 1-methyl-2-pyrrolidone (NMP) (99.5 vol%). The chemicals were purchased from Sigma Aldrich and used as received without any further purification. Ultra-pure water was used as the principal nonsolvent solution in the coagulation bath of the phase inversion method.

### 2.2. Methods

Real water samples were sampled in the Gauteng and KwaZulu Natal provinces of South Africa using 1L glass bottles with Teflon lined caps. In Gauteng, raw water samples were collected at Florida Lake (FL) and a water treatment plant that purifies water from the Vaal dam (RVD). In KwaZulu Natal, inlet raw water samples were collected at the Hazelmere water treatment plant which purifies water from the Hazelmere dam (HZ). Water samples were collected using the grab water sampling approach, transported to the UNISA Florida science campus in Johannesburg and stored at 4 °C until further use.

The membranes reported in this work were prepared through phase inversion. The polymer (PES) was initially dried in an oven at 120 °C for 12 h to remove moisture. The dried polymer was cooled, weighed and added to a weighed mixture of PEG and NMP. The solutions were stirred at approximately 60 °C for at least 14 h at 350 rpm to ensure complete dissolution of the polymer. The solution was later kept at room temperature to completely degas. A casting knife set at 150 µm thickness gap and a glass plate was used to cast the solution before immersing into a coagulation bath containing the nonsolvent solution. The prepared membranes were kept in ultra-pure water at 4 °C prior to characterization. Table 1 shows the polymer solution composition and the composition of the coagulation bath used to delay the rate of solvent–nonsolvent demixing during membrane preparation.

The morphology of the prepared membranes was determined using scanning electron microscopy (SEM) (JSM-IT300 Joel, Tokyo, Japan). To study the cross-sectional morphology, the membranes were cut into smaller pieces and dipped in liquid nitrogen for fracturing. Fractured membrane pieces where then coated with gold (5 nm) using a sputter coater (Quorum Q150R ES, Darmstadt, Germany) and transferred to the microscope for examination.

The topology and roughness of the membranes were examined using atomic force microscope (AFM, Alpha30 WITec focus innovations, Ulm, Germany). Pieces of the prepared membranes were cut and dried for 12 h at 40 °C. The dried membranes were cut into small pieces and mounted on glass slide using a two-sided glue tape. A maximum of three measurements from different points of each membrane were taken at a scan area of 50 µm by 50 µm using the non-contact mode of surface analysis. Surface roughness measurements of the membranes are specified in terms of the mean roughness (Sa) and the root mean square of the data (Sq).

Contact angle measurements for all the membranes prepared were performed using the DSA30E Kruss drop shape Analyzer, GmbH, Hamburg, Germany. Contact angle measurements were meant to determine the wettability of the prepared membranes. The membrane samples were initially dried for 12 h at 40 °C, then cut into small pieces, attached on a glass slide and analyzed using the sessile drop method. A syringe with deionized water was selected and used for all the measurements. The contact angle was measured using a minimum of 10 water drops at different spots on each membrane surface. Measurements for each drop were recorded twice and the average readings were reported. Contact angle (θ) measurements are generally used to determine the surface free energy through the Young–Dupre equation [14], which links the contact angle of a drop of a liquid placed on a flat solid surface with the surface tension of a liquid [14,15].

The mechanical properties of the membranes were determined using the SAXSpace system (Anton Paar GmbH, Graz, Austria). The membranes were dried at 40 °C for 24 h and dimensions of 40 mm length and 10 mm width were cut and attached to a sample holder. The cross-section area of the membranes was calculated by multiplying the width and the thickness measured using a digital micrometer. Each sample was clamped on the sides of an aluminum tensile stage and stretched at a speed of 0.50 mm/min. Three samples were tested, and the average was reported for tensile strength and elongation.

Membrane porosity measurements were determined through the dry–wet method. Membranes that were initially stored in deionized water were removed and placed between a paper towel to remove excess water. The membranes were then weighed to measure the weight of wet membrane (*W_w_*). The wet membrane was then dried for 24 h under vacuum at 60 °C followed by weighing for dry membrane (*W_d_*). Membrane porosity was then evaluated through Equation (1).

(1) $$\varepsilon \left(\%\right)=\frac{W_{w}-W_{d}}{\rho_{w}A\delta }\times 100$$

where ε is membrane porosity, $W_{W}$ and $W_{d}$ is the weight of the wet membrane and dry membrane, respectively (g). $\rho_{w}$ is the density of water (g/cm^3^), A is the effective membrane area (cm^2^) and δ is thickness of the membrane (cm). 

Pure water and synthetic/real water flux was measured in triplicates using a crossflow system at a pressure of 3 bar. The membranes were cut into 8.6 cm length and 3.9 cm width dimensions (effective membrane area = 0.00354 m^2^) and assembled within the crossflow pressure cells. The membranes were first compacted at 7 bar for 1 h to allow for the attainment of a steady flux before collecting the permeate. The water flux was calculated using Equation (2) [16].

(2) $$J_{w}=\frac{V}{\Delta t\cdot A}$$

where *J_w_* is water flux (L·m^−2^·h^−1^), V is the permeate volume (*L*), Δt is the time taken to collect the permeate (h) and *A* is the effective membrane area (m^2^).

Rejection studies were performed using a dead-end system at 3 bar. A synthetic feed solution of bovine serum albumin (BSA) (30 mg/L) was prepared by dissolving the pure substance in distilled water. A UV/Vis Spectrometer (Lambda 650 S, Waltham, Massachusetts, United States of America) was used to determine the UV absorbance of BSA at 280 nm in the feed and permeate solution. The UV absorbance in the feed and permeate solutions was used to calculate percentage rejection achieved by the membrane through Equation (3) [17].

(3) $$R\left(\%\right)=\frac{C_{f}-C_{p}}{C_{f}}\times 100$$

where R(%) is solute rejection, $C_{f}$ is the feed concentration and $C_{p}$ is the permeate concentration.

Antifouling propensity of the prepared membranes was investigated using synthetic water (HA, FA and BSA) and real water samples. The membranes were backwashed with deionized water at 7 bar for 30 min after filtration of feed water. Thereafter, the flux of the backwashed membrane was measured under the initial operating conditions (3 bar). The flux recovery (FRR) was evaluated using Equation (4).

(4) $$FRR\;\left(\%\right)=\frac{J_{w2}}{J_{w1}}\times 100$$

where $J_{w2}$ is the flux of backwashed membrane and $J_{w1}$ is the initial flux of the membrane prior to backwashing.

## 3. Results

### 3.1. Morphological Studies

**Figure 1:**
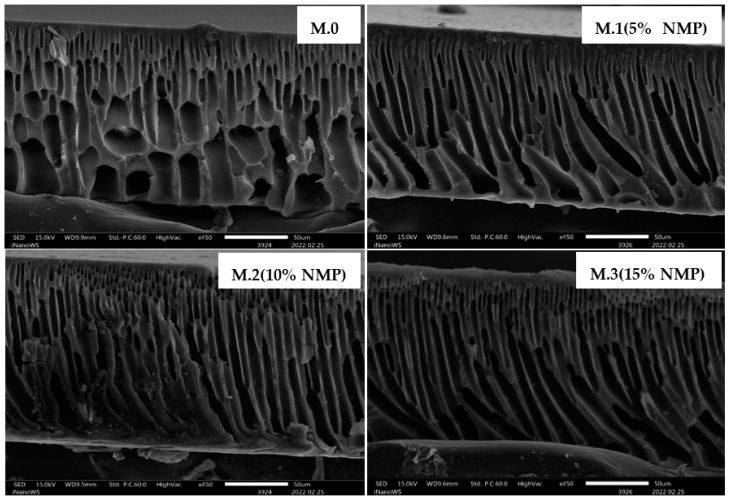
Scanning electron microscopy cross-sectional images of membranes prepared through delayed rates of solvent–nonsolvent demixing.

The SEM cross-sectional images of membranes prepared through delayed solvent–nonsolvent demixing are shown in Figure 1. All membranes prepared exhibit the finger-like morphology which commonly produces high water permeabilities and low solute rejections [9]. Moreover, a delay in the rate of solvent–nonsolvent demixing did not significantly alter the cross-sectional morphology of the membranes, i.e., the finger-like morphology was maintained even for membranes prepared through delayed solvent–nonsolvent demixing. It has been reported [12,18] that the addition of a solvent in the coagulation bath delays phase inversion and favors the formation of sponge-like membrane morphology. Liu et al. [18] and Xu et al. [12] demonstrated that increased concentrations of solvent in the coagulation bath slows down the rate of non-solvent diffusion into the polymer solution and suppresses the formation of finger-like structures. In this study, the presence of the solvent in the coagulation bath produced linear, narrow and continuous macrovoids indicating that the solvent in the coagulation bath is not the determining factor for the development of a spongy morphology. Porosity data on Table 2 supports the insignificant morphological changes on Figure 1 as the difference in porosities are also negligible. The cross-sectional image of M.0 shows short macrovoids, whereas membranes prepared through slower rates of phase inversion have linear, narrow and continuous macrovoids. Furthermore, the number of such narrow channels seems to increase with increasing concentration of solvent in the coagulation bath.

Several studies have used hydrophilic substances such as carbon nanotubes to increase miscibility between polymer solution and nonsolvent (water) in the coagulation bath, thereby increasing the rate of phase inversion [5,19,20,21]. Their results demonstrated the creation of a finger-like morphology from a sponge-like morphology. Strathmann et al. [10] postulated that finger-like membrane morphologies can be formed when the rate of the nonsolvent influx exceeds the solvent outflow. This study showed that coagulation bath composition can also be used to prepare specific membrane morphologies, which therefore disdains the use of costly, toxic and unstable nanoparticles for this objective [8,13,22,23].

**Figure 2:**
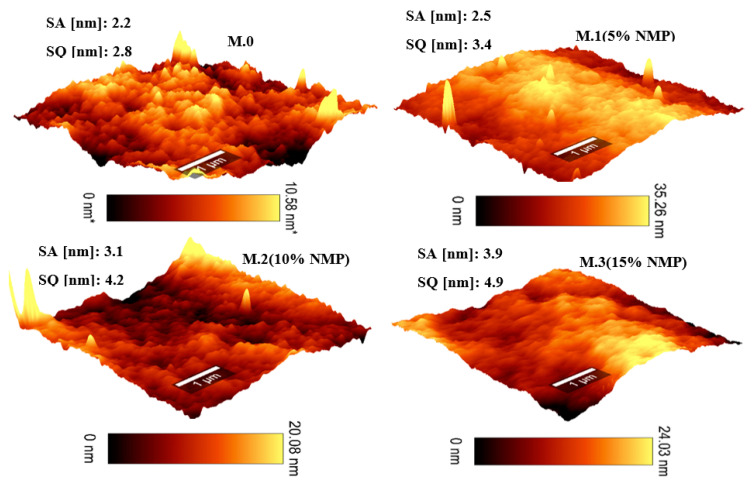
Atomic force microscopy topographical 3D images and surface roughness values of membranes prepared through delayed rates of solvent–nonsolvent demixing.

Surface properties of the membrane were studied using AFM and are displayed in Figure 2 as 3D topography images and surface roughness values. It can be seen that the root mean square roughness (SQ) and roughness average (SA) values of membranes prepared through delayed rates of phase inversion are higher than that of the pristine membrane. This can be attributed to the slow exchange between the solvent and nonsolvent which results in the retainment of high PEG concentrations in the membrane matrix. PEG is a viscous (poor mobility) and hydrophilic substance. Higher concentrations of PEG in the membrane matrix result in rough and hydrophilic surfaces [5]. It can be seen on Figure 2 that surface roughness values increase with a decreasing rate of solvent–nonsolvent demixing.

Mahlangu et al. [24] reported that an increase in membrane roughness translates to an increase in the membrane’s effective area due to ridges and valleys on the surface which are beneficial for flux enhancement, solute rejection and fouling resistance. In addition to the formation of long and narrow morphological channels shown on Figure 1, this study also demonstrates that delayed phase inversion leads to the formation of rough membrane surfaces.

### 3.2. Contact Angle (Wettability)

**Figure 3:**
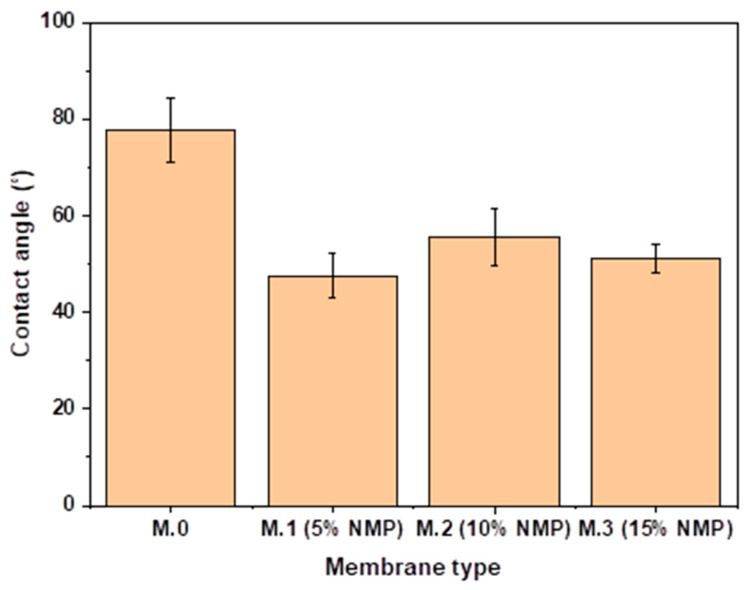
Contact angles of pristine membrane and membranes prepared through delayed solvent–nonsolvent demixing.

Contact angle measurements were performed to show the membrane’s surface hydrophilicity/wettability. Generally, a membrane is considered hydrophilic when the contact angle is less than 90° [25]. It can be seen on Figure 3 that upon delaying solvent–nonsolvent demixing, the contact angle decreased from 77.6° for pristine membrane (M.0) to 47.6° for M.1 (5%NMP), suggesting an enhancement in hydrophilicity. Xu et al. [12] also reported enhanced hydrophilicity of membranes due to delayed solvent–nonsolvent demixing. Liu et al. [18] and Ahmad et al. [26] also reported that the addition of a solvent in the coagulation bath delayed liquid–liquid demixing and improved membrane hydrophilicity. The enhanced hydrophilicity is due to the entrapped high PEG content in the membrane matrix as a result of the slow nonsolvent and PEG diffusion. However, the contact angles of membranes on Figure 3 are only slightly different after delaying solvent–nonsolvent demixing, suggesting that only a small concentration of solvent is required in the coagulation bath to decrease the contact angle, thereby enhancing hydrophilicity. The respective contact angles of the membranes prepared through delayed solvent–nonsolvent demixing are 47.6° (M.1(5%NMP)), 55.6° (M.2(10%NMP)) and 51° (M.3(15%NMP)).

In addition to morphological and surface roughness changes, this study also demonstrated that delayed solvent–nonsolvent demixing as a result of the addition of solvent in the coagulation bath enhances the membrane’s hydrophilicity, which is beneficial for flux and fouling resistance.

### 3.3. Mechanical Properties

**Figure 4:**
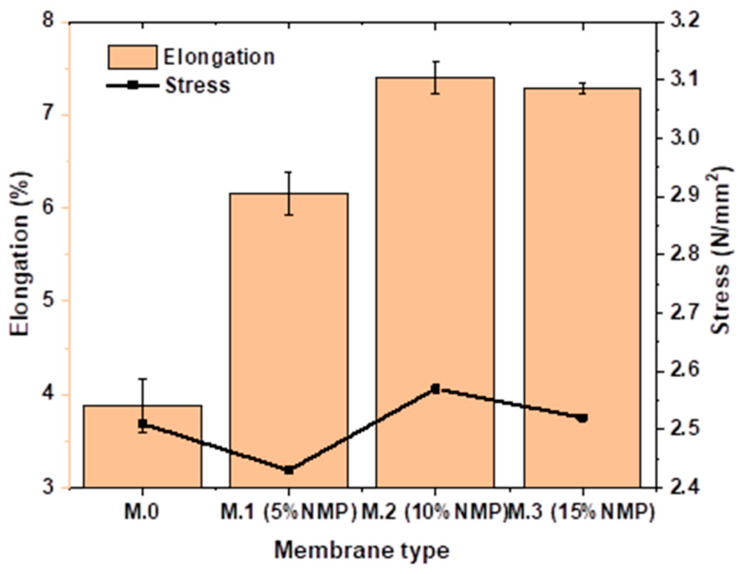
Tensile strength and elongation of pristine membrane and membranes prepared through delayed solvent–nonsolvent demixing.

A good membrane must resist physical damage during operation under external pressure. The mechanical strength properties of the membranes prepared in this study have been expressed in terms of tensile strength (N/mm^2^) and elongation at break (%). It can be seen in Figure 4 that delayed solvent–nonsolvent demixing increases elongation at the break of the membranes. Membrane M.0 recorded a tensile strength of 2.51 N/mm^2^ and an elongation at break of 3.9%. At 2.57 N/mm^2^ and 2.52 N/mm^2^, Membrane M.2 and M.3 recorded elongation at break of 7.4% and 7.3%, respectively. These data show that delayed solvent–nonsolvent demixing increases the elasticity of membranes, thereby increasing resistance to break. This result is attributed to decreased porosity and a morphology shift towards sponge-like morphology as a result of delayed solvent–nonsolvent demixing. Macrovoids and the finger-like morphology have been reported to be mechanically weak points that are susceptible to rupture/breaking when an external force is applied [5].

The tensile strength of M.0 is 2.51 N/mm^2^, while the tensile strength of M.2 with the highest elongation at break is 2.57 N/mm^2^. The insignificant effect on tensile strength is related to the low effect of this technique on membrane morphology (Figure 1).

### 3.4. Performance: Flux and Rejection

**Figure 5:**
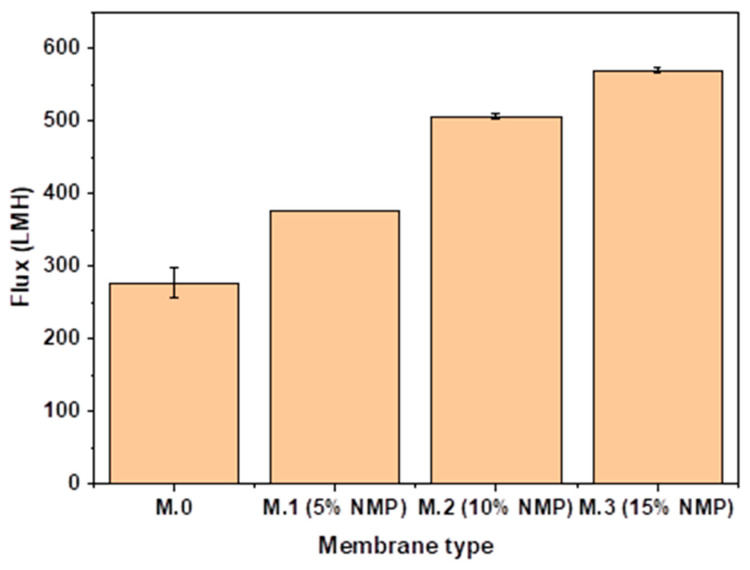
Pure water flux of pristine membrane and membranes prepared through delayed solvent–nonsolvent demixing.

**Figure A1:**
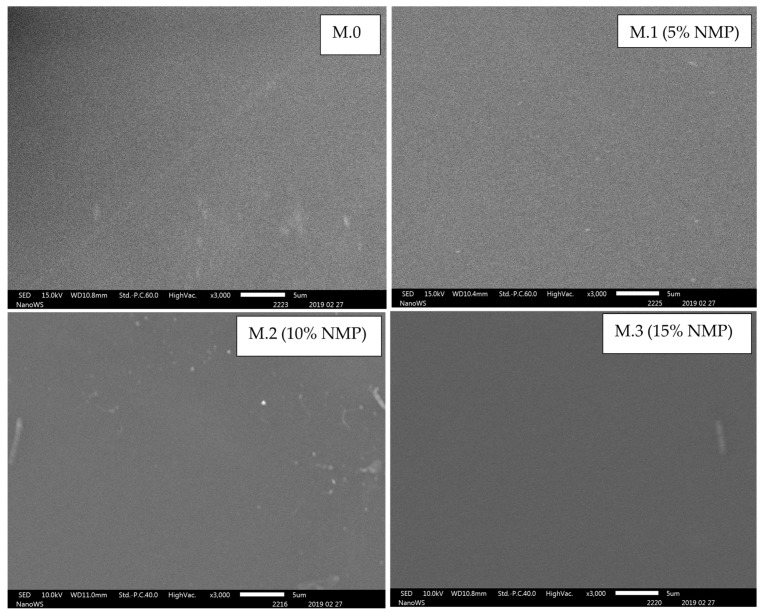
Scanning electron microscopy surface images of membranes prepared through delayed rates of solvent–nonsolvent demixing.

The fabricated membranes were evaluated for their performance regarding pure and synthetic/real water flux. It can be seen in Figure 5 that pure water flux increased with increasing concentration of solvent in the coagulation bath. Enhanced pure water flux for membranes prepared through delayed solvent–nonsolvent demixing is attributed to improved hydrophilicity (Figure 3) and continuous long narrow voids, as seen in Figure 1. The continuous long narrow channels in Figure 1 enhance the transport of permeating water due to less tortuosity and accommodation of high flow rates. High water permeation through carbon nanotube-embedded membranes is generally also attributed to the smooth inner walls of the nano material that offers less resistance to permeating water [27,28,29]. Surface pore size distribution is also a critical factor during ultrafiltration. Figure A1 shows surface images of membranes prepared in this study. However, no clear correlation can be seen between flux and pore distribution. Performance comparison of membranes prepared in this study and nanoparticle embedded UF membranes shows that our membranes produce a pure water flux comparable to that of nanoparticle-embedded UF membranes. Table 3 shows the pure water flux of membranes prepared in this study and that of nanoparticle-embedded membranes prepared in other studies.

It can be seen on Table 3 that nanoparticle-embedded UF membranes produce a pure water flux that is comparable to that of membranes fabricated in this study. However, nanoparticle-embedded membranes usually operate at low pressures (1 bar). The pure water flux of membranes prepared in this study was obtained at 3 bar, which is still in the ultrafiltration range. Drawbacks associated with performance optimization through incorporating nanoparticles in the membrane matrix include costly preparations, poor stability and the production of nanoparticle-contaminated water [13,34,38]. It is also well established that nanoparticles have a negative impact on various biological organs such as the reproductive system [8,38,39,40]. This known toxicity of nanoparticles still hinders the commercialization of nanoparticle-embedded membranes to date. It is therefore appropriate to rely on other strategies such as coagulation bath composition for UF performance enhancement.

Table 4 shows the removal of BSA by M.3 (15%NMP) and nanoparticle-embedded UF membranes. It can be seen that most nanoparticle-embedded UF membranes achieve high BSA rejections of greater than 95%. This study’s most permeable membrane (M.3 (15%NMP)) produced a BSA Rejection of 88%, which is comparable/within reach to performances of nanoparticle-embedded UF membranes. High removal of BSA is an admirable membrane property since it indicates the ability of the membrane to remove the persistent protein fraction of NOM, which is difficult to remove through upstream techniques such as coagulation and flocculation [41].

### 3.5. Fouling Trends and Antifouling Tests

#### 3.5.1. Normalized Flux and Fouling

**Figure 6:**
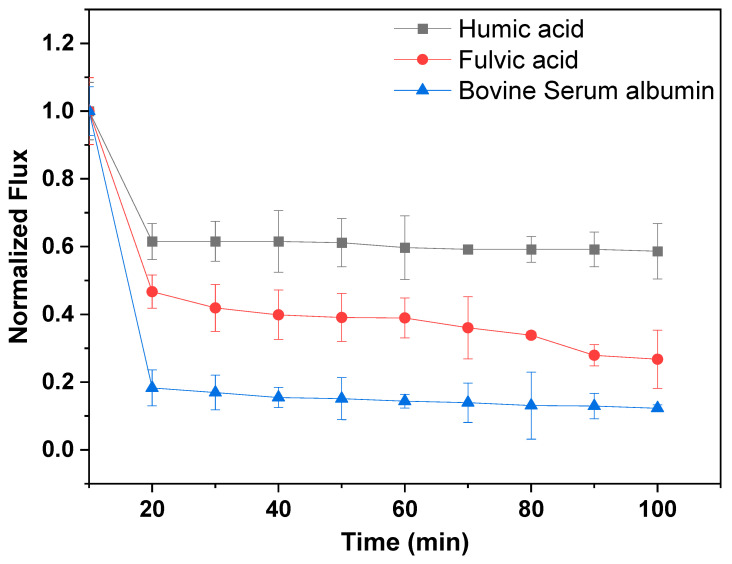
Normalized flux of M.3 (15%NMP) during filtration of synthetic humic acid, fulvic acid and bovine serum albumin water.

**Figure 7:**
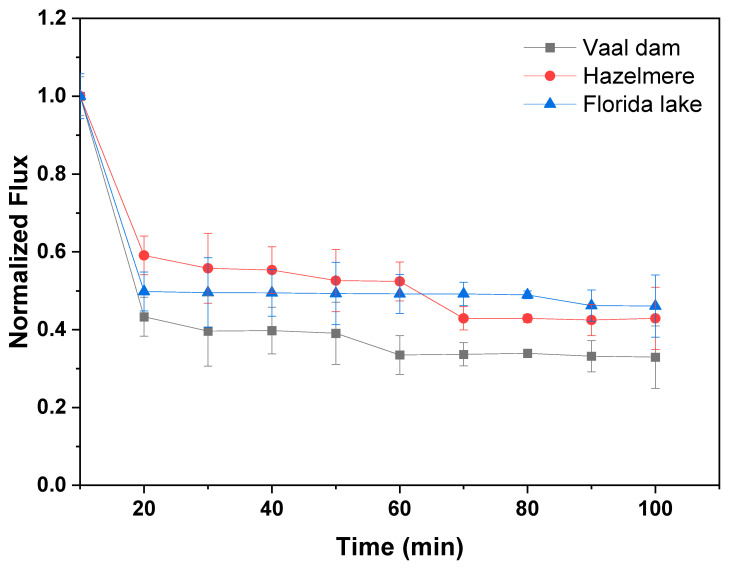
Normalized flux of M.3 (15%NMP) during filtration of real water samples.

Normalized flux of the most permeable membrane (M.3 (15%NMP)) with regard to synthetic and real water samples is shown in Figure 6 and Figure 7, respectively. It can be seen in Figure 6 that M.3 (15%NMP) fouls significantly when exposed to feed water concentrated with BSA and fulvic acid. The membrane lost 80% and 50% of its permeability after 20 min of filtration with BSA and fulvic acid feed water, respectively. Both BSA and fulvic acid are considered as polar/hydrophilic substances, which means that they both enter and foul the membrane matrix as water permeates through (pore blockage fouling) [44]. However, fouling studies were performed with highly concentrated synthetic water samples (30 mg/L) for worst case scenario tests. Furthermore, at water treatment plants, membrane filtration units are applied after feed water pretreatment for fouling alleviation. It can therefore be assumed that fouling will not be severe with real water samples and pretreated water. These data also show that feed water concentrated with the fulvic acid and protein fraction (BSA) of NOM has a high potential for low permeability and severe membrane fouling. Water quality parameters of real water samples are presented in Table 5. It can be seen in Figure 7 that real water samples also induce a sharp flux decline, which can also be reduced through feed water pretreatment.

Vaal dam water samples have the highest aromatic content (High SUVA and UV_254_), hence the low flux and quickest flux decline due to the rejection of hydrophobic aromatic substances and the formation of a cake layer on the membrane surface [38]. It is clear from Table 5 and Figure 7 that water samples with the highest aromatic content induced the most severe flux decline on our most permeable membrane, i.e., Vaal dam > Hazelmere > Florida lake.

**Figure 8:**
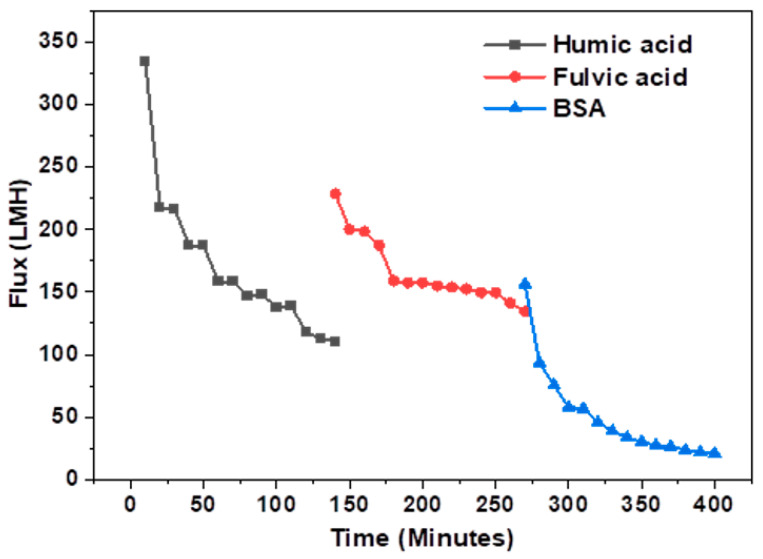
Flux decline of M.3(15%NMP) after filtration with synthetic water samples.

Fouling studies were also performed through sequential filtration of synthetic water samples without washing the membrane between cycles. It can be seen in Figure 8 that severe membrane fouling is induced by BSA. BSA is a polar substance that was 88% rejected by the membrane (Table 4). Nevertheless, because of its polarity and its affinity for water and the membrane surface, BSA still enters the membrane matrix and blocks membrane pores. This type of fouling is severe, difficult to reverse and sometimes irreversible [42]. These data show that a high rejection of BSA is crucial for produced water quality and fouling alleviation. The membrane maintained a flux of above 100 LMH with humic acid and fulvic acid feed water. This shows that fouling through humic and fulvic acid is mostly in the form of a lose cake layer that has little effect on permeation [41]. Furthermore, it can be expected that humic and fulvic acid fouling will be reversible through backwashing since it is on the surface of the membrane and not in the membrane matrix.

#### 3.5.2. Flux Recovery

**Figure 9:**
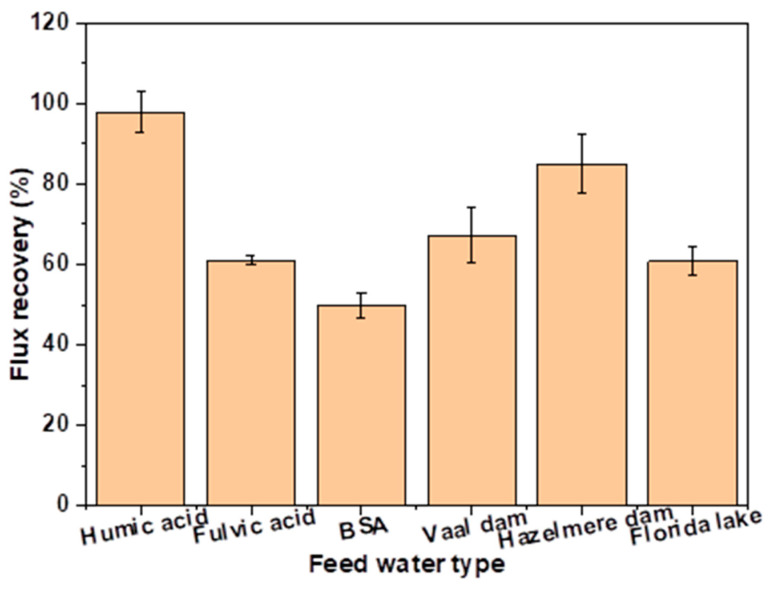
Flux recovery of M.3 (15%NMP) after the filtration of different feed water samples.

The antifouling propensity of the most permeable membrane (M.3 (15%NMP)) was evaluated through flux recovery (FRR) analysis. It is clear in Figure 9 that the membrane has the highest and the lowest FRR after the filtration of humic acid and BSA, respectively. BSA is a polar substance that has an affinity for permeating water and the membrane surface. Therefore, some fouling by BSA or protein substances can be inside pores and irreversible [41]. Humic acid is hydrophobic and causes membrane fouling mostly through a cake layer which is easy to remove through backwashing, hence the high FRR by M.3 (15%NMP) after backwashing with distilled water. Membranes developed through the incorporation of nanoparticles have demonstrated high FRR (>90%) after filtration of BSA feed water [5]. M.3 (15%NMP) showed comparable surface hydrophilicity and BSA rejection with nanoparticle-incorporated membranes, which therefore means that the antifouling property of nanoparticle-embedded membranes is due to other properties besides surface hydrophilicity. In the future, this study will use other solutions such as alkali, surfactants and oxidants for backwashing in order to cost-effectively clean the membrane and achieve high FRR.

This study notes that nanoparticle-embedded membranes are the future of membrane science. Therefore, this study is not meant to discourage the incorporation of nanoparticles on membranes because of the associated drawbacks. However, as much as researchers move into nanoparticle-embedded membrane research, interest in simple/plain cost-effective polymeric membranes must continue together with the search for effective membrane cleaning solutions.

## 4. Conclusions

This work studied the feasibility of membrane performance optimization through a delayed solvent–nonsolvent demixing preparation technique. It was found that delayed solvent–nonsolvent demixing influences membrane properties such as morphology, porosity, surface roughness, hydrophilicity and elasticity, thereby showing suitability for alterations and membrane performance optimization. The finger-like morphology of the membrane did not significantly change with delayed rates of phase inversion. However, macrovoids evolved to be long, linear and narrow as an effect of a delayed phase inversion. Surface hydrophilicity and roughness increased with decreasing rates of solvent–nonsolvent demixing due to increased content of entrapped PEG in the membrane matrix. Delayed rates of phase inversion enhanced the membrane’s performance and produced a comparable pure water flux and BSA rejection with nanoparticle-incorporated membranes. BSA was found to be the NOM fraction that induces severe membrane fouling. Furthermore, fouling studies showed that membranes prepared in this study have low backwashing flux recovery when compared to nanoparticle-embedded membranes. It is therefore recommended that future studies should look into cost-effective physical and chemical membrane cleaning methods so that flux recovery and membrane lifespan can be enhanced.

## Figures and Tables

**Table 1:** Compositions of the polymer solutions and coagulation bath (Sol/Nsol *v*/*v*.%).

Membrane	PES wt.%	NMP wt.%	PEG wt.%	Sol/Nsol *v*/*v*.%
M.0	20	44	36	0/100
M.1 (5% NMP)	20	44	36	5/95
M.2 (10% NMP)	20	44	36	10/90
M.3 (15% NMP)	20	44	36	15/85

**Table 2:** Thickness and porosity of membranes prepared in this study.

Membrane	Thickness (µm)	Porosity (%)
M.0	221 ± 4.9	77 ± 2.5
M.1 (5% NMP)	229 ± 3.4	73 ± 3.6
M.2 (10% NMP)	214 ± 4.1	67 ± 4.1
M.3 (15% NMP)	211 ± 4.7	70 ± 2.7

**Table 3:** Pure water flux of membranes prepared through delayed solvent–nonsolvent demixing and nanoparticle-embedded membranes.

Membrane	Pure Water Flux (LMH)	Reference
M.0	276 @ 3 bar	This work
M.1 (5% NMP)	376 @ 3 bar	This work
M.2 (10% NMP)	505 @ 3 bar	This work
M.3 (15% NMP)	568 @ 3 bar	This work
SPES-ZnO-g-PSPA	420 @ 1 bar	[4]
M-MWCNT	578 @ 1 bar	[5]
PES	102 @ 3 bar	[30]
PES	170 @ 3 bar	[31]
COOHC1.3PS85PV13.2	71 @ 3 bar	[32]
PSF-PVP/LDH-M	592 @ 1 bar	[33]
PVDF-HNTs	315 @ 1 bar	[34]
PVDF-TiO_2_/HNTs	263 @ 1 bar	[35]
PSU/GO	309 @ 1 bar	[36]
PES-HNTs	454 @ 1 bar	[37]

**Table 4:** Rejection of BSA by UF membrane prepared in this work and nanoparticle-embedded UF membranes.

Membrane	BSA Rejection (%)	Reference
M.3 (15%NMP)	88%	This work
PES/SPSF/O-MWCNT	100%	[5]
CA/PVP/TiO_2_	97%	[42]
SIMo/PS/PSf	99%	[6]
PSF/GO	95%	[7]
PVDF/CuXO/GO	80%	[43]
PVDF/TiO_2_/GO	89%	[6]

**Table 5:** Water quality parameters of real water samples.

Parameter	Florida Lake	Vaal Dam	Hazelmere
DOC (mg/L)	7.3	1.2	2.9
UV_254_ (cm^−1^)	0.2	0.7	0.2
SUVA (L/mg·m)	3.2	62.1	6.4
Conductivity µS/cm	615	498	418
pH	7.3	6.9	7.1

## Data Availability

The data set presented in this work forms part of a bigger project with proprietary information and can be availed on request.
